# Positive verbal suggestion optimizes postural control

**DOI:** 10.1038/s41598-019-42888-2

**Published:** 2019-04-23

**Authors:** Bernardo Villa-Sánchez, Mehran Emadi Andani, Giulia Menegaldo, Michele Tinazzi, Mirta Fiorio

**Affiliations:** Department of Neurosciences, Biomedicine and Movement Sciences, University of Verona, Verona, Italy

**Keywords:** Cognitive neuroscience, Human behaviour

## Abstract

Balance is a very important function that allows maintaining a stable stance needed for many daily life activities and for preventing falls. We investigated whether balance control could be improved by a placebo procedure consisting of verbal suggestion. Thirty healthy volunteers were randomized in two groups (placebo and control) and asked to perform a single-leg stance task in which they had to stand as steadily as possible on the dominant leg. The task was repeated in three sessions (T0, T1, T2). At T1 and T2 an inert treatment was applied on the leg, by informing the placebo group that it was effective in improving balance. The control group was overtly told that treatment was inert. An accelerometer applied on participants’ leg allowed to measure body sways in different directions. Subjective parameters, like perception of stability, were also collected. Results showed that the placebo group had less body sways than the control group at T2, both in the three-dimensional space and in the anterior-posterior direction. Furthermore, the placebo group perceived to be more stable than the control group. This study represents the first evidence that placebo effect optimizes posture, with a potential translational impact in patients with postural and gait disturbances.

## Introduction

The placebo effect is a beneficial outcome that follows the administration of a treatment and that is not to be ascribed to active ingredients but to the words, contexts and beliefs that surround the treatment and that can induce psychological and neuronal changes in the recipient’s brain^1^. Accumulating evidence suggests that the placebo effect extends beyond pain. To date, many behavioural studies have demonstrated the efficacy of placebos in influencing aspects of motor performance like speed, force, and resistance to fatigue in athletes, non-athletes and patients with motor symptoms^2–9,10^. Motor performance is a multifaceted definition that refers to several different dimensions, including not only speed, force, and resistance to fatigue, but also precision control, motor skill learning and balance, among others. Knowledge on the potential effect of placebos on these aspects of motor performance is still lacking. In the present study, we aimed at investigating whether the placebo effect can influence a very important component of human motor functions, like balance control. Balance allows to maintain a stable and upright stance needed for many daily life activities and for preventing falls. Human stance is not a static but a dynamic phenomenon^11,12^, characterized by small instabilities called body sways. Controlling posture through balance requires a complex organization and interaction between motor coordination and sensory systems, such as the somatosensory, vestibular and visual systems^13,14^.

The cerebral cortex has an important role for human balance control^15–17^, as demonstrated using corticomuscular coherence^18,19^, electroencephalography^20,21^ and neuroimaging^22^. Moreover, TMS studies showed enhanced corticospinal excitability in standing balance compared to nonstanding or supported conditions, hinting at a role of the corticospinal system in this function^23,24^. Crucially, the placebo effect in the motor domain appears to increase the excitability of the corticospinal system^7^, thus giving neurophysiological support to the potential link between the placebo effect and balance control.

In the present study, we adopted a parallel design in which a single-leg stance task was executed by two groups of healthy participants (placebo and control). The placebo group received the application of an inert electrical device over the leg muscle together with verbal information about its positive effects on balance. The control group received the application of the same device with overt information about its inefficacy in changing balance. To measure balance, we developed a new custom-made user-friendly device consisting of a sensor attached to the leg that easily allows a fine-tuned detection of body sways in different directional planes, without any platform or unwieldy equipment. This feature makes it practically useful in future studies aiming at exporting this paradigm outside the laboratory to improve balance control in patients affected by postural deficits or in gait disorders in which the pharmacological treatment is often ineffective. We hypothesized that subjects of the placebo group would enhance balance control after the procedure compared to the control group.

## Methods

### Participants

The sample size was computed with G-Power 3.1^25^. Considering a standard effect size (f) of 0.25 (which is considered as medium according to Cohen^26^), power (1-β error probability) of 0.8, α error probability of 0.05, correlation among repeated measures of 0.5, the resulting sample size is 28. To avoid a reduction of statistical power due to potential dropouts, thirty healthy participants were recruited (14 females; mean ± SD: 20.1 ± 1.3 years) from the student population of the University of Verona. Participants were divided in two different groups matched for sex, age, height and foot size: 15 subjects (7 females; mean age: 20 ± 1.4 years; mean height: 172.3 ± 10.8 cm; mean foot size: 26.5 ± 1.9 cm) were enrolled in the placebo group, and 15 subjects (7 females; mean age: 20.1 ± 1.1 years; mean height: 171.4 ± 8.8 cm; mean foot size: 26.3 ± 1.8 cm) were enrolled in the control group. Height and foot size were matched between groups in order to rule out any influence of these two variables on the balance motor task. Participants were all dominant on the right lower limb, according to the Edinburgh questionnaire^27^. Participants gave their written informed consent at the beginning of the experiment and were debriefed about the placebo nature of the study only after completing the whole experimental procedure. The study was conducted in accordance with the declaration of Helsinki and was approved by the committee for approval of research on humans (CARU) of the University of Verona.

### Single-leg stance task

Subjects were asked to stand on the floor as steadily as possible with the dominant leg for 30 seconds while keeping the arms along the body. The non-dominant leg was kept in suspension with the knee flexed. This task consisted of 10 trials and was performed in three experimental sessions (as described in detail below). The first 3 seconds of each trial were used to calculate the subject’s initial upright position. This value served as reference to trace the amount of leg displacements during the rest of the trial.

Inspired by Dejnabadi *et al*.^28^, subjects’ movements were recorded by means of a custom-made three-dimensional accelerometer (ADXL345) placed on the dominant lower limb and connected to a microcontroller board. In the range of low frequency movements like normal gait and single-leg stance task, the joint angles can be measured accurately using accelerometer^29–31^. This system allows to achieve a proper and high-resolution detection of subjects’ movement sways (with a precision of 0.04 degrees). Moreover, the system is characterized by high flexibility, being made by a friendly device easy to apply, to remove and to transport. These features make the system a potential instrument t o easily analyse balance outside the laboratory, such as in the clinical setting for the study of patients with postural deficit.

Data were stored for offline analysis with Matlab (Matlab 2014, MathWorks). After each single trial, there was a rest of 30 seconds, in which participants were allowed to stand on both legs. During the task, a PC monitor was placed in front of the participants at a distance of 100 cm and served to give the instructions and as fixation frame during the trials. To rule out any confounding effect related to the type of shoe on balance control, participants were barefoot during the task.

### Procedure

**Figure 1:**
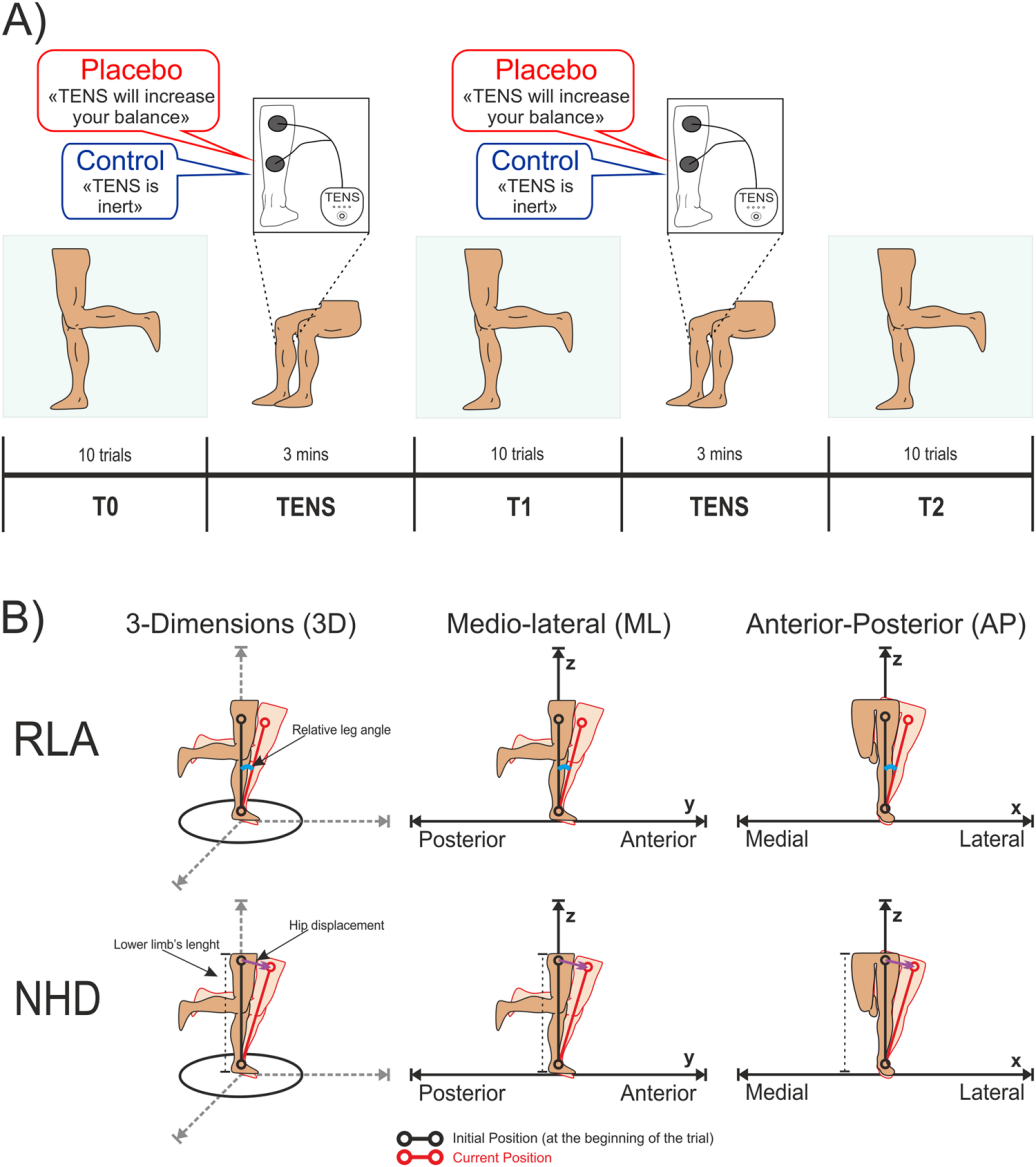
(**A**) Schematic representation of the experimental protocol. The procedure consisted of three sessions (T0, T1 and T2). In each session, participants performed a single-leg balance task by standing as steadily as possible with the dominant leg for 30 seconds. The non-dominant leg was kept in suspension with the knee flexed. Participants repeat the described task 10 times for each session. Before T1 and T2, transcutaneous electrical nerve stimulation (TENS) treatment was applied on the leg while subjects were seated for 3 minutes. Different verbal information about the effects of TENS was given to the placebo and control group. (**B**) Representation of the behavioural indexes of balance. Relative leg angle (RLA) was computed as the angular deviation (in degrees) of the dominant leg during the trial (red line on the leg) with respect to the initial upright position measured in the calibration (black line on the leg). Maximum values of RLA were taken in the three-dimensional space (RLA-3D), in the medial-lateral direction (RLA-ML) and the anterior-posterior direction (RLA-AP). Normalized hip displacement (NHD) was defined as the displacement of the subject’s hip during the trial (red lines on the leg) with respect to the initial position (black lines on the leg) normalized to the length of the subject’s lower limb. Maximum values of NHD were taken in the three-dimensional space (NHD-3D), in the medial-lateral direction (NHD-ML) and the anterior-posterior direction (NHD-AP). The figure illustrates the movement in one direction as an example; actual body sways, instead, could have occurred in both directions and dimensions.

At the beginning of the experimental procedure, participants performed a training of 2 trials (15 seconds each) to familiarize with the task. During the experiment, the balance task was performed three times: T0, T1, T2 (Fig. 1A). T0 was considered as baseline and allowed to obtain a measure of performance before any manipulation. T1 and T2 allowed to measure any change in performance after the first and second application of the placebo treatment, respectively.

The placebo treatment consisted in the application of transcutaneous electrical nerve stimulation (TENS) on one of the muscles involved in the balance task, specifically the grastrocnemius muscle of the dominant leg for 3 minutes while participants were seated. The intensity of TENS was adjusted until subjects felt a slight sensation on the skin without muscle contraction. The frequency of TENS was set at 10 Hz and was completely inefficient in inducing any active modification of balance performance. Nonetheless, participants of the experimental group were told that TENS had the effect of increasing the recruitment of muscle fibers and consequently of improving balance control. TENS was applied twice (T1 and T2) to investigate for potential strengthening of the placebo effect. Participants of the control groups underwent the same procedure, but with different verbal information about TENS. Specifically, they were clearly told that they entered a control group in which TENS was to be applied with an inactive mode.

### Measures of performance

#### Behavioural parameters

Leg movements and spatial position recorded through the accelerometer were considered as proxy of balance control. In particular, we derived two main parameters: relative leg angle (RLA) and normalized hip displacement (NHD). RLA is defined as the angular deviation (in degrees) of the dominant leg with respect to its initial upright position measured at the beginning of each trial (Fig. 1B). To characterise the subject’s movements in a fine-tuned way, different indexes were derived from RLA. Specifically, we measured the maximal postural sway defined as the maximum RLA obtained in the three-dimensional space (RLA-3D). High values of RLA-3D are indicative of large postural sways. We also derived the total amount of movement variability defined as the standard deviation of RLA in the three-dimensional space (RLA-3D_std_). Higher values of RLA-3D_std_ are indicative of higher postural variability. Finally, we derived the body sways in specific directional planes, such as the maximum RLA in the medial-lateral direction (RLA-ML) and the maximum RLA in the anterior-posterior direction (RLA-AP).

The NHD parameter consisted of the displacement of the subject’s hip (HD in cm) with respect to the initial position, normalized to the length of the subject’s lower limb, i.e. NHD = HD/(leg length) × 100.

Similar to RLA, several indices were extracted to deeply analyse subject’s movements. NHD-3D is defined as the maximum NHD obtained in the three-dimensional space and NHD-3D_std_ is defined as the NHD standard deviation in the three-dimensional space. In addition, NHD-ML and NHD-AP were also measured to better characterize movement directions in the medial-lateral and anterior-posterior directions, respectively (Fig. 1B).

For all the indexes, the median of the 10 trials was calculated in each session; the median is a more robust estimator of the central value and is less sensitive to outliers. Higher values in these indexes indicate worse balance control.

#### Subjective parameters

Subjective variables were also evaluated throughout the procedure. Particularly, at the end of each session after having completed the balance task participants were asked to judge how stable they have felt on a 10 cm visual analogue scale (VAS) ranging from 0 (very unstable) to 10 (very stable). This gave us a measure of the subjective perception of stability. To carefully monitor the subjective sense of extent throughout the experimental procedure, we asked participants to complete the Borg scale, ranging from 0 (rest) to 10 (maximal effort)^31,32^ after each trial in each session. Expectation about the effects of TENS was measured soon after each TENS application (before task execution) by asking participants to judge whether they expected an improvement or worsening of performance on a number rating scale (NRS) ranging from −3 (much worse than at baseline) to +3 (much better than at baseline), with 0 (the same as at baseline). The perception of treatment efficacy was measured after completion of the balance task at T1 and T2, by asking participants to judge whether TENS was effective or not in enhancing stability on a 10 cm VAS ranging from 0 (not effective at all) to 10 (extremely effective).

### Statistical analysis

Behavioural (RLA-indexes and NHD-indexes) and subjective parameters (perception of balance, sense of effort, expectation, perception of treatment efficacy) were analysed using SPSS Statistics 21 software (IBM SPSS Statistics 21, SPSS Inc., Chicago, IL). Normality of data distribution was checked with the Shapiro-Wilk test and the z-score transformation was applied before running parametric tests if normality was violated. More precisely, z-scores were calculated for each group separately using the following formula:

$${{\rm{Z}}}_{{\rm{i}},{\rm{n}}}=({{\rm{X}}}_{{\rm{i}},{\rm{n}}}-{{\rm{M}}}_{{\rm{T}}0})/({{\rm{SD}}}_{{\rm{T}}0}).$$

where, i is the value at T0, T1 or T2, n is the subject number, M_T0_ and SD_T0_ are the mean value and standard deviation of the index at T0 session.

Behavioural data were analysed by means of repeated measures analysis of variance (ANOVA), with Group (placebo, control) as between-subjects factor and Session (T0, T1, T2) as within-subjects factor.

Ordinal data, like the subjective data, were analysed with non-parametric tests. Precisely, the Mann-Whitney U test was applied to compare the two groups (Placebo vs. Control) in each session separately (T0, T1, T2). The test of Friedman was used to analyse the factor Session (T0, T1, T2) within each group separately.

In the case of significant factors, post hoc comparisons were carried out with paired samples and independent samples t-test for parametric analysis and with Wilcoxon signed-rank test for non-parametric analysis.

The Cohen’s *d* statistic was used to analyse the effect size of all the results^25^. Bonferroni correction for multiple comparisons was applied when needed and the level of significance was set at *p* ≤ 0.05.

## Results

Height and foot size were matched between groups in order to rule out any influence of these two variables on the balance motor task. The statistical analysis confirmed that the two groups did not statistically differ for height (*p* = 0.810) or foot size (*p* = 0.800).

### Relative leg angle (RLA)

**Figure 2:**
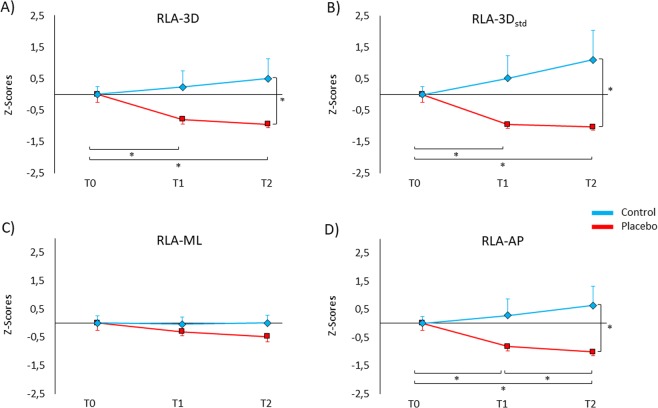
Z-scores of the behavioural data for the relative leg angle (RLA). Positive values represent more postural sways, negative values represent less postural sways. (**A**) RLA-3D (in the three-dimensional space) was lower in the placebo group (red line) compared to the control group (blue line) at T2. Moreover, participants of the placebo group showed lower postural sways at T1 and T2 compared to T0. (**B**) Similarly, RLA-3D_std_ was lower in the placebo group compared to the control group at T2. Additionally, RLA-3D_std_ was lower at T1 and T2 compared to T0 in the placebo group. (**C**) RLA-ML (in the medial-lateral direction) showed no significant differences between groups and across sessions. (**D**) RLA-AP (in the anterior-posterior direction) was lower in the placebo group than the control group at T2. In the placebo group, RLA-AP was lower at T2 compared to T1 and T0, and at T1 compared to T0. Values are expressed as z-scores ± SE. *p < 0.050.

Being RLA indexes not normally distributed (Shapiro-Wilk, p < 0.050), data were submitted to the z-score transformation before entering into the analysis. Analysis of RLA-3D revealed a significant Group × Session interaction (F_(2,56)_ = 5.72, *p = *0.005). Post-hoc comparisons showed that in the placebo group postural sways were lower at T1 (−0.79 ± 0.16) and at T2 (−0.94 ± 0.12) compared to T0 (0.0 ± 0.26) (for both comparisons, *p* < 0.015; Cohen’s d, d > 1.20), whereas in the control group no significant effect was found across sessions (T1: 0.23 ± 0.52, T2: 0.50 ± 0.64, *p* > 0.843). Moreover, the placebo group had lower sways than the control group at T2 (*p* = 0.036, d = 0.81). This finding indicates that after the experimental procedure, the placebo group was overall more able than the control group to control the postural sways in the three-dimensional space (Fig. 2A). No significant effect was found for the factor Session (*p = *0.408) and Group (*p = *0.093).

With regards to the standard deviation, analysis of RLA-3D_std_ showed a significant Group × Session interaction (F_(2,56)_ = 5.67, *p = *0.006). Post-hoc comparisons showed that in the placebo group RLA-3D_std_ values were lower at T1 (−0.95 ± 0.12) and T2 (−1.03 ± 0.09) compared to T0 (0.0 ± 0.26) (for both comparison, *p* = 0.006, d > 1.35), while values of the control group did not differ across sessions (T1: 0.51 ± 0.74; T2: 1.10 ± 0.97; *p* > 0.150). Moreover, participants of the placebo group had lower RLA-3D_std_ values than the control group at T2 (*p* = 0.038, d = 0.79) (Fig. 2B). No significant effect was found for the factor Session (*p = *0.696) and Group (*p = *0.072).

No significant effect was found for RLA-ML with regard to Session (*p = *0.217), Group (*p = *0.399) and Group × Session interaction (*p = *0.232) (Fig. 2C).

The analysis of RLA-AP disclosed a significant Group × Session interaction (F_(2,56)_ = 5.84, *p = *0.005). Post-hoc comparisons showed that the placebo group had lower RLA-AP values at T1 (−0.81 ± 0.16) and at T2 (−1.02 ± 0.13) than at T0 (0.0 ± 0.26) (for both comparisons, *p* < 0.018, d > 1.17) and at T2 compared to T1 (*p* = 0.042, d = 1.01), whereas in the control group no significant effect was found across sessions (T1: 0.28 ± 0.59, T2: 0.64 ± 0.70, *p* > 0.315). Moreover, RLA-AP values were lower in the placebo group compared to the control group at T2 (*p* = 0.029, d = 0.84). This finding confirms a better performance of the placebo group and adds a specificity for a better control of body sways in the anterior-posterior direction (Fig. 2D). No significant effect was found for the factor Session (*p = *0.541) and Group (*p = *0.083).

### Normalized hip displacement (NHD)

**Figure 3:**
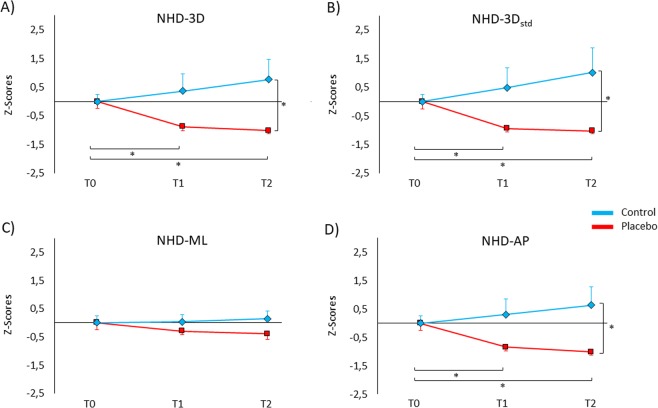
Z-scores of the behavioural data for the normalized hip displacement (NHD). Positive values represent more postural sways, negative values represent less postural sways. (**A**) NHD-3D (in the three-dimensional space) was lower in the placebo group (red line) compared to the control group (blue line) at T2. Additionally, participants of the placebo group showed lower postural sways at T1 and T2 compared to T0. (**B**) In the same way, NHD-3D_std_ was lower in the placebo group compared to the control group at T2. Moreover, NHD-3D_std_ was lower at T1 and T2 compared to T0 in the placebo group. (**C**) NHD-ML (in the medial-lateral direction) revealed no significant differences between groups and across sessions. (**D**) NHD-AP (in the anterior-posterior direction) was lower in the placebo group than the control group at T2. In the placebo group, RLA-AP was lower at T2 and T1 compared to T0. Values are expressed as z-scores ± SE. *p < 0.050.

Being NHD indexes not normally distributed (Shapiro-Wilk, p < 0.050), data were submitted to the z-score transformation before entering into the analysis. Overall, the findings related to the NHD are in line with those of the RLA index. More precisely, the analysis of NHD-3D revealed a significant interaction Group × Session (F_(2,56)_ = 5.72, *p = *0.005). *Post hoc* comparisons showed lower NHD-3D values in the placebo group at T1 (−0.87 ± 0.15) and T2 (−1.01 ± 0.12) than at T0 (0.00 ± 0.26) (for both comparisons, *p* < 0.018, d > 1.16), whereas in the control group no significant effect was found across sessions (T1: 0.36 ± 0.62; T2: 0.76 ± 0.73; *p* > 0.234). Moreover, NHD-3D values of the placebo group were significantly lower compared to the control group at T2 (*p* = 0.025, d = 0.86) (Fig. 3A). No significant effect was found for the factor Session (*p = *0.636) and Group (*p = *0.066).

Analysis of NHD-3D_std_ disclosed a significant effect of the Group × Session interaction (F_(2,56)_ = 6.01, *p = *0.004). *Post hoc* comparisons revealed that placebo group had lower NHD-3D_std_ values at T1 (−0.94 ± 0.12) and T2 (−1.02 ± 0.0) compared to T0 (0.00 ± 0.25) (for both comparisons, *p* < 0.018, d > 1.31), whereas in the control group no significant effect was found across sessions (T1: 0.48 ± 0.71; T2: 1.00 ± 0.90; *p* > 0.123). Moreover, NHD-3D_std_ values were lower in the placebo group compared to the control group at T2 (*p* = 0.032, d = 0.82). No significant effect was found for the factor Session (*p = *0.697) and Group (*p = *0.069) (Fig. 3B).

Analysis of NHD-ML did not reveal significant effects for Session (*p = *0.659), Group (*p = *0.315) and Group × Session interaction (*p = *0.247) (Fig. 3C).

Analysis of NHD-AP revealed a significant interaction Group × Session was also significant (F_(2,56)_ = 5.90, *p = *0.005). *Post hoc* comparisons revealed that the placebo group had lower NHD-AP at T1 (−0.87 ± 0.15) and at T2 (−1.01 ± 0.12) than at T0 (0.00 ± 0.25) (for both comparisons, *p* < 0.018, d > 1.13), whereas in the control group no significant effect was found across sessions (T1: 0.31 ± 0.57; T2: 0.63 ± 0.68; *p* > 0.402). Moreover, the placebo group had lower NHD-AP values than the control group at T2 (*p* = 0.025, d = 0.86). No significant effect was found for the factor Session (*p = *0.545) and Group (*p = *0.068) (Fig. 3D).

### Subjective parameters

**Figure 4:**
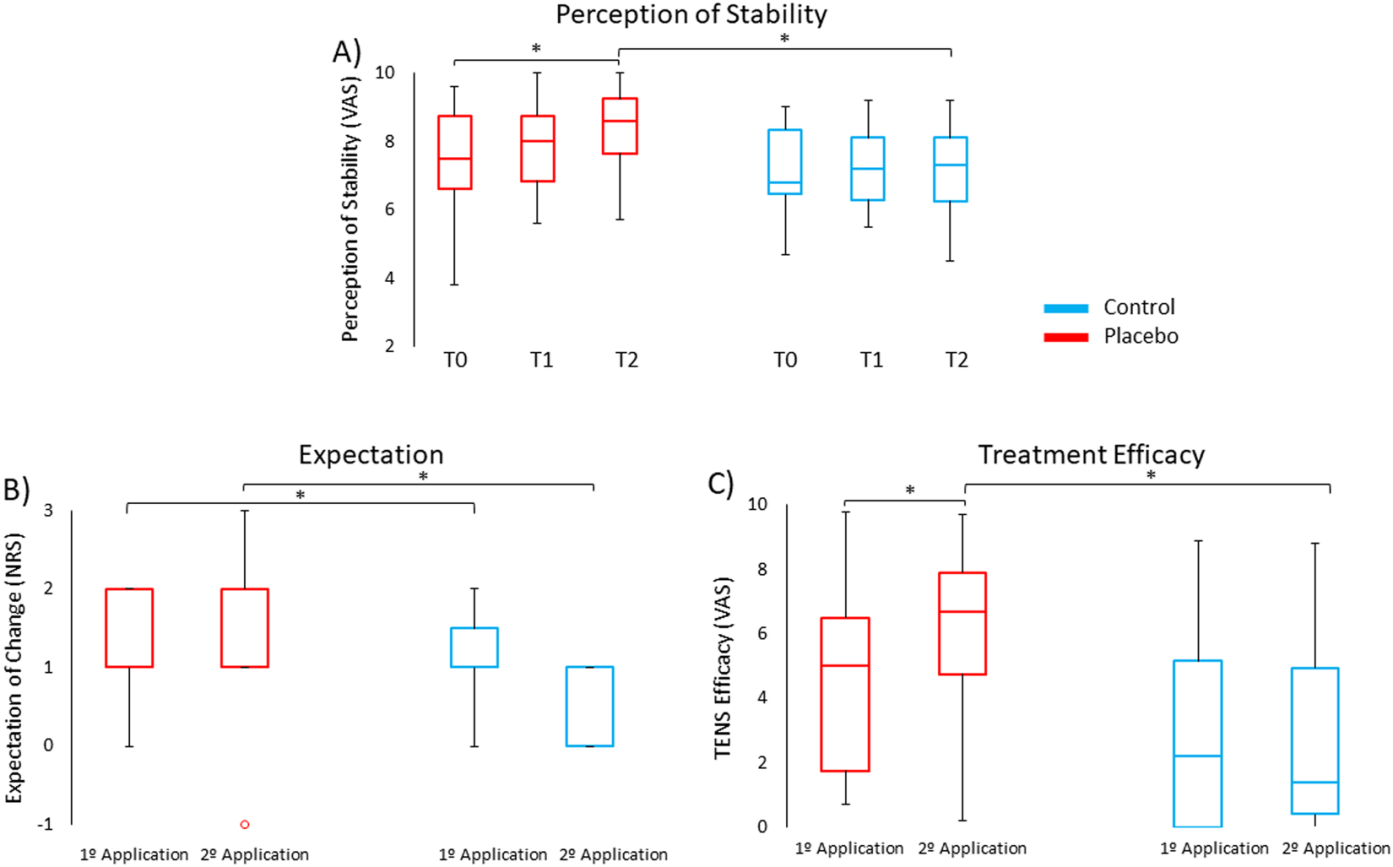
Box plots of the subjective data in the placebo group (red) and in the control group (blue). (**A**) Perception of stability in the placebo group at T2 was higher compared to T0 and compared to the control group. (**B**) Subjects of the placebo group expected to be more stable compared to subjects of the control group after both TENS applications. (**C**) Subjects of the placebo group perceived more effect of TENS after the second than the first application. Moreover, after the second application subjects of the placebo group perceived more effects of TENS than the control group. *Significant values (p < 0.05); °outliers (values that were located outside 1.5 times the interquartile range above the upper quartile and below the lower quartile) were included in the non-parametric analysis; Horizontal lines represent median values.

Regarding the perception of stability, the Mann-Whitney test revealed that subjects of the placebo group (median ± interquartile range, Mdn ± IQR = 8.6 ± 1.8) perceived higher stability than the control group (Mdn = 7.3 ± 2.0) at T2 (U = 60.0, *p* = 0.029, d = 0.87), whereas the groups did not differ at T0 and T1 (*p* > 0.16). This finding suggests that participants of the placebo group not only were more stable than the control group but also had a subjective perception of more stability. The Friedman test resulted significant for the placebo group (*χ*^2^ = 10.4, *p* = 0.005) and not for the control group (*p* = 0.94). Post-hoc comparisons indicated that subjects of the placebo group perceived themselves more stable at T2 (Mdn = 8.6 ± 1.8) compared to T0 (Mdn = 7.5 ± 2.3) (Z = 2.75, *p = *0.018, d = 1.15) (Fig. 4A).

The analysis of the sense of effort (Borg) did not show significant differences between groups in any of the three sessions (*p* > 0.96). Similarly, the Friedman test revealed no significant difference within groups (*p* > 0.88). This suggests that the task induced the same perception of effort in the two groups and therefore the different ability to control body sways as well as the different perception of stability were not related to more or less fatigue in performing the task.

The analysis of expectation showed that participants of the placebo group (Mdn = 1.0 ± 1.0) had higher expectation of improvement than participants of the control group (Mdn = 0.0 ± 1.0) soon after the first TENS application (U = 26.0, *p* < 0.001, d = 1.96). Similar results were obtained after the second TENS application (U = 42.0, *p* = 0.001, d = 1.42), as subjects of the placebo group expected more improvement (Mdn = 1.0 ± 1.0) than those of the control group (Mdn = 0.0 ± 1.0) (Fig. 4B). These findings suggest that the verbal suggestion used in the placebo procedure was successful in inducing a positive expectation of improvement.

The Mann-Whitney test indicated that subjects of the placebo group perceived the TENS as more effective (Mdn = 6.7 ± 3.3) than the subjects of the control group (Mdn = 1.4 ± 4.6), when they were asked at the end of T2 (U = 51.2, *p = *0.01, d = 1.03). No difference in TENS efficacy scores was found between groups at the end of T1 (*p = *0.08). The Wilcoxon test showed significant differences in the placebo group (Z = 2.41, *p* = 0.016, d = 0.98), with higher TENS efficacy scores after T2 (Mdn = 6.7 ± 3.3) than T1 (Mdn = 5.0 ± 5.1). No differences were found for the control group (*p* = 0.58). This result suggests that the perception of TENS efficacy increased during the experiment in the placebo group, demonstrating that the placebo manipulation worked successfully (Fig. 4C).

## Discussion

The aim of this study was to investigate whether the placebo effect could be induced in a monopodalic balance task. Our findings reveal for the first time that balance control can be improved with a placebo procedure consisting of verbally-induced positive expectations about the efficacy of a treatment.

Our findings show that participants of the placebo group had an overall decrease of body sways in the three-dimensional space compared to the control group, hinting at a better postural control. The improvement of balance was found consistently in the indexes extracted from the relative leg angle and the normalized hip displacement. More precisely, the values of RLA and NHD of the placebo group were lower than those of the control group specifically at T2. This may suggest that repeated exposure to the treatment could induce stronger placebo effects, as shown in previous studies adopting conditioning procedures^5,33,34^. It should be noted, however, that in our study the effects of the treatment were only verbally suggested, without conditioning. Finding a reduction of body sways in the placebo group at T1 and T2 compared to T0 indicates that verbal suggestion alone could improve balance control from the very first application of the treatment and this improvement remained stable.

The improvement of balance found at a general level (i.e., 3D space) in the placebo group was present for both indexes also in the AP (but not in the ML) direction. These findings are in line with the notion that body sways in the AP and ML directions represent different components of postural stability and also require different cortical activation to be adequately controlled^21,35^. Typically, sways in the AP direction are bigger in magnitude than sways in the ML direction^35–37^. This pattern seemed to be present also in our study and it could explain why a within-group reduction from T0 to T1 to T2 could more easily emerge in the AP direction. We could speculate that the placebo group developed a specific postural strategy during the placebo procedure. According to the optimal control model^38^, the choice of the postural strategy depends on the postural goal and on the environmental constraints. In our study, the postural goal was to maintain the body aligned to the upright position. In this type of task, as well as in unperturbed stance^39,40^, the ankle strategy consisting of ankle torque, seems to be privileged to control posture^39^. Interestingly, balance in the AP direction is under ankle control^41–43^. Hence, the specific reduction of body sways we found in the AP direction could suggest that the placebo group adopted a postural strategy based on the ankle in order to better control balance.

Displacements in the ML direction, which are under hip control^41–43^, have been more often associated to higher postural instability, with consequent risk of falls, and have been related to task difficulty both in healthy individuals and in pathological populations^35,37,44,45^. Of note, the control of balance in the ML direction may be more important in older than in young adults^46^ and this could explain why we did not find any effect in the ML direction in our young healthy subjects.

With regard to the subjective variables, we found that perception of stability was in line with the behavioural results, in that participants of the placebo group perceived to be more stable across sessions and compared to the control group. This suggests that the placebo procedure could modulate both behavioural and subjective parameters^47,48^. Expectations scores of the placebo group were significantly higher compared to the control group after the two applications of the TENS treatment, suggesting that the verbal information about TENS was effective in inducing positive expectations of better performance. Perception of TENS efficacy was also higher in the placebo group, indicating that participants of the placebo group believed in the efficacy of the TENS.

The application of the inert TENS treatment to both groups allows to discard any influence on balance control due to the mere leg stimulation. The lack of improvement in the control group may be ascribed to the difficulty of the monopodalic task, which tends to induce higher postural instability than bipedal tasks^49,50^. Due to the absence of difference between groups in the BORG scale, the subjective sense of effort does not seem to have influenced the performance at the task.

Overall, our findings suggest that balance control and perception of stability can be improved by a placebo procedure.

The mechanisms underneath these findings have not been investigated in our study, although it is reasonable to speculate that the placebo-induced improvement of balance could be related to the involvement of the brain network involved in this motor function. Postural control is a complex motor skill based on the communication between different sensorimotor systems^51^ and on the activity of cortical and subcortical brain regions^15,17,20^. Classically, postural control has been thought to be mainly regulated by subcortical regions^52^. More recent studies support a different conception that hints at the involvement of the cerebral cortex^15,20^. In particular, two main circuits seem to be important for postural control: a cortical-cerebellar loop and a cortical-brainstem loop involving the basal ganglia^15,53–55^.

Studies with electroencephalography and transcranial magnetic stimulation suggest that a set of cortical regions may be involved in different aspects of postural control, like the primary motor cortex, the supplementary motor area, the cingulate cortex, the parietal, temporal and insular cortex^15,56–61^. These regions concur in the integration of sensory input (mainly vestibular and somatosensory) needed for optimal postural control, as well as in the pre-selection and optimization of postural responses needed to anticipatorily control an eventual loss of balance^56,62–66^. Interestingly, some of these regions can be activated also by the motor imagery of a balance task combined with action observation^67^, hinting at the possibility of deploying these approaches to modulate balance control. Moreover, electrophysiological studies have revealed the crucial role of motor cortical areas and of the corticospinal system in balance control^24,61,68^. Of note, postural responses involve the activation of muscle synergies throughout the entire body^15,69–72^ and the motor cortex plays an important role in activating these muscle synergies^73–75^. A previous study with transcranial magnetic stimulation showed that a placebo procedure could influence the activity of the primary motor cortex, enhancing the excitability of the corticospinal system^7^. Hence, based on this evidence we speculate that our placebo intervention could have optimized the corticospinal control of muscle synergies, thus resulting in better balance performance.

Brain regions involved in anticipatory postural control could also play a role. With regard to this, it was demonstrated that anticipatory postural adjustments are associated with the activity in three brain networks, including not only the somatosensory-motor network but also the cingulo-opercular and the fronto-parietal networks that could exert a top-down control on posture^76^. Moreover, anticipated postural perturbations have been associated to changes in the readiness potential^15,59^. The readiness potential is an electrophysiological sign of cortical excitability recorded before voluntary movement onset and seems to be generated in the sensory-motor cortex and in the supplementary motor area^77,78^. An earlier study revealed that the readiness potential could be modulated by placebo procedures and this modulation was associated to the placebo-induced reduction of fatigue^8^. Hence, it could also be suggested that the placebo intervention in our study influenced the brain regions involved in the anticipatory control of posture in order to better prevent a potential loss of balance.

Our task was not based on the use of perturbations to measure subjects’ postural control, but on a continuous intentional control of balance in a monopoladic stance. This type of task most likely involves voluntary control and cortical centres^23,24,79,80^. Hence, based on the abovementioned studies, we would suggest that cortical regions involved in voluntary postural control could play a role in the placebo-induced enhancement of balance. This hypothesis should be proved in future studies by applying neurophysiological techniques.

Previous studies have demonstrated that placebos can improve different aspects of motor performance, such as speed, resistance to fatigue and force^2,7,9^. This study represents the first behavioural evidence that a placebo procedure in the motor domain can also improve balance control and perception of stability. These findings can have important translational relevance for clinical populations (like for instance Parkinson’s disease) and for the elderly, in which balance disturbances increase the risk of falls with a consequent negative impact on the quality of life. Moreover, improving balance through a placebo procedure could have a beneficial impact on gait disorders in which the pharmacological treatment is often not effective. Future neurophysiological investigations are needed to uncover the precise mechanisms underpinning the placebo-induced enhancement of balance.

## Data Availability

The data generated and analysed during the current study are available from the corresponding author on reasonable request.
